# First Report of *Haemoproteus* (Haemosporida, Haemoproteidae) Megalomeronts in the Brain of an Avian Host, with Description of Megalomerogony of *Haemoproteus Pastoris*, the Blood Parasite of the Common Starling

**DOI:** 10.3390/ani11102824

**Published:** 2021-09-27

**Authors:** Mélanie Duc, Mikas Ilgūnas, Monika Kubiliūnaitė, Gediminas Valkiūnas

**Affiliations:** Nature Research Centre, Akademijos 2, 08412 Vilnius, Lithuania; ilgunasmikas@gmail.com (M.I.); moncelotas@gmail.com (M.K.); gediminas.valkiunas@gamtc.lt (G.V.)

**Keywords:** haemosporidian parasites, birds, *Haemoproteus pastoris*, exo-erythrocytic development, megalomeronts, brain

## Abstract

**Simple Summary:**

Birds are hosts to diverse blood parasites belonging to many taxonomic groups. Among them, numerous haemosporidian parasites of the genus *Haemoproteus* are transmitted globally. These pathogens develop in the blood and internal organs of birds. The blood stages (gametocytes) are known for about 150 described species, but the tissues stages or exo-erythrocytic stages (meronts and megalomeronts) are known only fragmentarily for about 10% of the described species. Knowledge on merogony is important in avian medicine for better understanding of pathologies during haemoproteosis. This study reported and characterized the megalomeronts of *Haemoproteus pastoris,* a parasite of the widespread Common starling (*Sturnus vulgaris*). Parasites were identified using molecular and microscopy examination tools. Five individual naturally infected birds were sampled, and their organs were examined histologically. Megalomeronts were found in eight different organs. The parasites were described and illustrated. The largest megalomeront, of all observed forms and shapes, reached 800 μm in length. Importantly, *Haemoproteus* megalomeronts were reported in the brain of avian hosts for the first time, indicating non-described pathology during avian haemoproteosis. This study contributes to a better understanding of the life cycle of avian haemoproteids and opens new perspectives in pathology research during avian haemoproteosis, which is important for birds’ health.

**Abstract:**

Species of *Haemoproteus* (Haemoproteidae, Haemosporida) are common bird pathogens. Recent molecular studies combined with histopathology research have reported development of megalomeronts of these parasites in various organs, sometimes resulting in the death of the avian host. Five Common starlings (*Sturnus vulgaris*) were found naturally infected with *Haemoproteus pastoris* lineage hLAMPUR01. The parasite was identified using microscopic examination of blood films and DNA sequences. Infected bird organs were investigated histologically for (i) the presence of exo-erythrocytic stages and (ii) the patterns of development (morphology and localization) in different host individuals. For the first time, megalomeronts of *Haemoproteus* parasites were seen developing in the brain, while numerous others at different stages of maturation were found in the intestine, pancreas, kidneys, lungs, esophagus, spleen, gizzard, and trachea. Megalomeronts were predominantly roundish or oval, up to 800 μm, they were surrounded by a capsular-like wall and developed asynchronously in the same bird individual. After megalomeront maturation and rupture, a massive infiltration of blood cells occurred, indicating the hemorrhagic processes. Review of available data showed that different *Haemoproteus* species produce markedly different megalomeronts, morphology of which can probably be predicted using phylogenetic analysis based on partial sequences of cytochrome *b* gene.

## 1. Introduction

Avian haemosporidians (Haemosporida, Apicomplexa) are endoparasites, which are transmitted by species of Diptera and develop in the blood cells and internal organs of vertebrates. Gametocytes of haemosporidians infect various blood cells, and their tissue stages or exo-erythrocytic stages (meronts and megalomeronts) develop in internal organs. Meronts are thin-wall structures about 50 μm in maximum length, while megalomeronts are big, usually roundish or oval structures often reaching more than 300 μm in diameter, surrounded by a thick-wall [1,2]. Haemosporidians are classified in four families: Plasmodiidae, Haemoproteidae, Garniidae, and Leucocytozoidae [1,2]. In terms of species and lineage diversity, *Haemoproteus* parasites of the Haemoproteidae outnumber the parasites found in other families (MalAvi database http://130.235.244.92/Malavi/ accessed on 28 September 2021 [2,3]). Haemoproteids are cosmopolitan and parasitize birds of the majority of orders, on all continents except Antarctica (MalAvi database [2,3,4,5]).

The Common starling (*Sturnus vulgaris*) is a common bird species found in Europe, where it is native and widespread. This species is markedly invasive and has been introduced in Asia, Australia, Northern America, and Northern and South Africa [6]. Being an omnivore bird species, it can be considered a pest due to flocking in gardens and agricultural lands in some countries [6]. Diversity of haemosporidian parasites is low in the Common starling, with four lineages of *Plasmodium* and only one species of *Haemoproteus*, *Haemoproteus pastoris* lineage hLAMPUR01, described in this naturally infected host (MalAvi database [3]). Available information about this pathogen covers only molecular data (partial cytochrome *b* gene sequence) [3], morphology of blood stages (gametocytes) [1], and sporogony in *Culicoides* biting midges [7,8]. Exo-erythrocytic development of *H. pastoris* remains unknown.

Indeed, research on exo-erythrocytic stages of *Haemoproteus* parasites is scarce, starting in 1908 with the discovery of exo-erythrocytic stages of *Haemoproteus columbae* in pigeons [9], followed by a few other reports of tissue stages in several different parasite species [10,11,12,13]. Due to the predominant opinion about harmlessness of avian *Haemoproteus* infections [14], the research on exo-erythrocytic stages of *Haemoproteus* spp. remained slow for over a century [15,16,17,18,19]. However, interest of researchers regarding this issue was renewed by reports providing molecular evidence about avian *Haemoproteus* infections, which are responsible for mortality in birds due to damage caused by tissue stages [20]. Several recent molecular studies supplemented with histopathology research have proved that *Haemoproteus* parasites influence the health of the infected bird [20,21,22,23,24], but the mechanisms of pathologies might be different in different pathogen species and remain poorly understood.

Several previous studies suspected occurrences of large megalomeront-like structures in birds during *Haemoproteus* infections [1,13], but there was no proof that they belonged to *Haemoproteus.* It was believed that the structures might have been stages of other apicomplexan parasites, for example, *Besnoitia* spp. [14]. Recently, molecular techniques have been applied in parallel with histology research in order to prove that megalomeronts certainly develop in host tissues during haemoproteosis, indicating that this developmental stage is an important part in the life cycle of *Haemoproteus* parasites [18,25,26]. However, patterns of megalomerogony remain unclear during haemoproteosis. According to the available information, tissue stages of *Haemoproteus* spp. have been found in the gizzard, heart, intestine, kidneys, liver, lungs, proventriculus, skeletal muscle, and spleen [13,22]. To date, they have not been found in the brain, nor the reproductive organs, but these organs can be parasitized by other haemosporidians. For example, phanerozoites of *Plasmodium* sp. develop in the brain and megalomeronts of *Leucocytozoon* sp. parasitize the brain and reproductive organs of birds [1,13]. Moreover, the few investigated species of *Haemoproteus* have shown a remarkable morphological diversity of megalomeronts, with cytomeres being separated by septa (*H. passeris* [13]) or not (*H. majoris* [22,26]) and megalomeronts developing in tight clusters (*H. minutus* [20], *H. asymmetricus* [25]) or not (*H. syrnii, H. velans* [21,23]). Several recent studies published detail colorful illustrations of developing and mature megalomeronts, including different stages of cytomeres formation and maturation of merozoites [22,24,25], while old publications have reported completely mature and ruptured megalomeronts for speculated *Haemoproteus* spp. [9,10,11,27]. Available limited information provides opportunities to start detailed comparative research on megalomerogony during avian haemoproteosis.

Further accumulation of data about exo-erythrocytic development of haemoproteids is needed. During this study, we sampled five Common starlings naturally infected with *Haemoproteus pastoris,* identified this parasite using microscopic examination and DNA barcoding sequences, and conducted histological examinations. We aimed to (i) determine the presence of exo-erythrocytic stages in this bird species during infection of one parasite lineage and (ii) gain information about the patterns of development (morphology and localization) in different host individuals. We also discussed available morphological information about megalomeronts of other *Haemoproteus* species in regard of their phylogenetic relationships.

## 2. Materials and Methods

### 2.1. Study Site and Samples

Common starlings were caught using permanent traps (large ‘Rybachy’ type, zigzag and funnel traps) and mist nets at the Ventes Ragas Ornithological station (55°20′38.93” N, 21°11′34.05” E), Lithuania in May 2019. This period corresponded to the beginning of the breeding season of Common starlings at the study site. Blood samples were collected by puncturing the branchial vein and fixed in SET-buffer [28] for further molecular analyses. A drop of fresh blood was used to prepare 2–13 blood films on ready-to-use glass slides. The films were fixed by immersion of the slides in absolute methanol for one second and then stained with 10% Giemsa [1]. In all, 19 Common starlings were sampled. Microscopic examination of blood films determined the presence of haemosporidians, and species of *H. pastoris* was identified [1]. Later, the species identification was confirmed by using molecular barcoding in the laboratory (see description below). Microscopic examination of blood films was performed using a BX61 light microscope (Olympus, Tokyo, Japan). Five microscopy-positive birds with gametocyte parasitemia between 1 and 26% of infected red blood cells (calculated according to [29]) were euthanized (by decapitation) and their organs were processed for histological examination.

### 2.2. Histological Examination

The brain, heart, intestine, pancreas, kidneys, liver, lungs, esophagus, pectoral muscles, spleen, gizzard, reproductive organs, and trachea were collected and fixed in 10% neutral formalin in the field. At the laboratory, the organs were processed using traditional histology techniques: dehydrating the samples in 96% EtOH, followed by a clarification in Isopropanol and embedded in paraffin blocks. For each organ, sections of 4 μm were prepared with a microtome, mounted on glass slides, air-dried, stained with hematoxylin–eosin (H&E) and covered with coverslip. Detailed histological procedures were described in [1,30].

All microscopic examinations of histological sections were performed in the laboratory using a light microscope BX41TF equipped with an Olympus DP12 digital camera and the image software Olympus DP-SOFT (Olympus, Tokyo, Japan). Each histological section was screened entirely at low (×200) and at medium magnification (×400). Depending on the organ size, about 0.5–1 mm depth of histological preparations was cut and examined. The highest magnification (×1000) was used to observe the structure of the megalomeronts. Different magnifications (×100, ×200, ×400, and ×1000) were used to prepare photographs aiming to better illustrate megalomeronts. The parasite measurements [diameter (for roundish-shape) and length x width (for oval-shape) of the megalomeronts, and thickness of capsular-like wall] were taken at most fitted magnifications, ×100, ×200, or ×1000 depending on the size of the object. Voucher preparations of parasite gametocytes (accessions 49,288 NS–49,319 NS) and megalomeronts (accessions 49,320 NS–49,360 NS) were deposited at the Nature Research Centre, Vilnius, Lithuania.

### 2.3. DNA Extraction, PCR and Sequencing

DNA (deoxyribonucleic acid) was extracted using an ammonium acetate protocol [31], and diluted in 1X TE buffer to work at a concentration of 25 ng/μL. A nested PCR (polymerase chain reaction) was used to determine the lineage of the parasite by amplifying 478 bp of the cytochrome *b* gene (cyt *b*), using the standard primers HaemNFI/HaemNR3 and HaemF/HaemR2 for *Haemoproteus* and *Plasmodium* parasites. The parameters of the nested PCR were as described in original protocols descriptions [28,32]. For the first amplification, the extracted DNA was used as a template. To control for possible contamination and false amplifications, a positive control (a *Haemoproteus* sp. positive sample), and a negative control (ddH_2_O) were used. Together with a dye, 2 μL of the final PCR product was run on a 2% agarose gel to check for positive amplification. Those were then sequenced from the 3′ end with a Big Dye Terminator V3.1 Cycle Sequencing Kit and ABI PRISM^TM^ 3100 capillary sequencing robot (Applied Biosystems, Foster City, CA, USA). Sequences were checked in Geneious Prime 2020.0.5 (https://www.geneious.com, accessed on 28 September 2021) for quality, identification of possible mixed infections (one peak for single infection, two or more peaks for mixed infections), and identification of lineage. The molecular results and blood film microscopy results were compared for species identification.

### 2.4. Phylogenetic Analysis

BLAST searches in MalAvi [3] and the NCBI GenBank (https://www.ncbi.nlm.nih.gov/genbank/, accessed on 28 September 2021) database were used for lineage identification using the obtained sequences. The following lineages sequences were retrieved from the GenBank database and used to build the phylogenetic tree: 30 lineages of *Haemoproteus* species, 6 of *Plasmodium* species, and 1 of *Leucocytozoon* species (lSISKIN2, as outgroup). Lineages of morphologically characterized parasites as well as *Haemoproteu*s spp. with described exo-erythrocytic development were incorporated into the analysis for comparative purposes. The software jModeltest-2.1.10 [33,34] was used to select the best-fit model (GTR+G+I), which was run in Geneious with the MrBayes plugin v3.2.6 [35] for 5 million generations, and sampled every 100th generation, while discarding the first 25% of trees as a ‘burn-in’ period for the construction of the consensus tree.

## 3. Results

### 3.1. Parasite Identification and Phylogenetic Relationships

**Figure 1:**
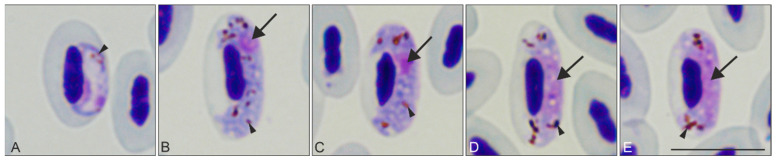
Gametocytes of *Haemoproteus pastoris* from naturally infected Common starlings *Sturnus vulgaris*. Young (**A**), growing (**B**,**D**), and mature (**C**,**E**) macrogametocytes (**B**,**C**) and microgametocytes (**D**,**E**) are shown. Note the presence of prominent pigment granules in gametocytes. The nuclei were compact and of sub-terminal position in macrogametocytes (**B**,**C**), but were diffuse and located centrally in microgametocytes (**D**,**E**). Ameboid extremities were visible in the growing gametocytes (**B**,**D**), and slight lateral displacement of the erythrocyte nuclei was seen in cells containing mature gametocytes (**C**,**E**). Triangle arrow (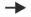)—parasite nucleus. Triangle arrowhead (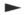)—pigment granules. Images were taken using Giemsa-stained blood films at ×1000 magnification. Scale bar = 10 μm.

Of the 19 examined Common starlings, seven (prevalence of 36.8%) were *Haemoproteus* sp.-positive by microscopic examination of blood films. Other blood parasites were not seen, except for *Lankesterella* sp. in individual no. 3 (Table 1). All reported *Haemoproteus* infections were *H. pastoris* based on gametocyte morphology (Figure 1). Five intensively infected birds, with parasitemia ranging between 1 and 26% (Table 1), were used for investigation of tissue stages. Gametocytes on various stages of development (young Figure 1A, growing Figure 1 B,D and mature Figure 1 C,E) were seen in these birds, indicating asynchronous gametocytogony.

**Figure 2:**
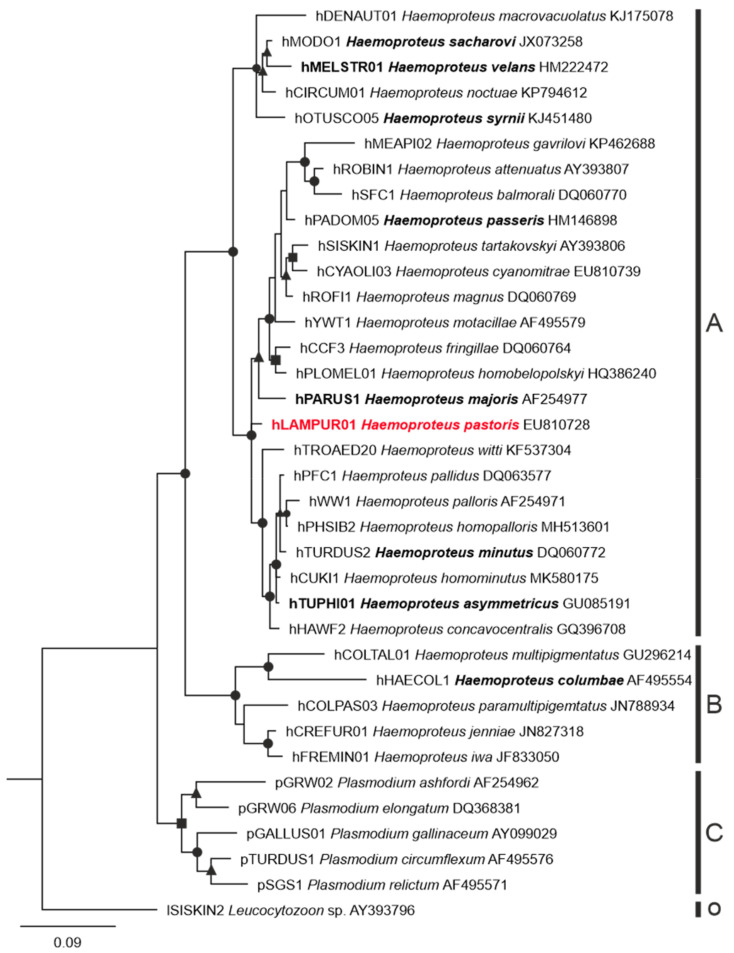
Bayesian phylogenetic tree of partial cytochrome *b* sequences of 30 lineages of *Haemoproteus* parasites, 6 lineages of *Plasmodium* spp., and 1 *Leucocytozoon* sp. lineage used as an outgroup. Vertical bars (A-O) represent parasite species of the subgenera *Parahaemoproteus* (**A**), *Haemoproteus* (**B**), parasites of the genus *Plasmodium* (**C**), and the outgroup, a *Leucocytozoon* sp. parasite (O). Bold font indicates names of *Haemoproteus* species and their corresponding lineage for which megalomeronts were previously reported; note that these parasites are present throughout the phylogeny and have markedly different megalomeront morphology (see Discussion section for further explanation). The parasite lineage studied in this work is given in red font. Posterior probabilities are provided with symbols: triangles—0.7–0.8; squares—0.8–0.9; rounds—0.9–1. Lineage names are given according to MalAvi database (http://130.235.244.92/Malavi/, accessed on 28 September 2021), followed by their parasite species names and sequence GenBank accession numbers.

Only the sequence of hLAMPUR01 lineage was identified in all dissected Common starlings for *Haemoproteus* parasites. This sequence clustered with the other sequences of haemoproteids belonging to subgenus *Parahaemoproteus* but was phylogenetically apart in relation to other *Parahaemoproteus* and *Haemoproteus* species for which megalomeronts are known (Figure 2). The sequence of pGRW11 of *Plasmodium relictum* was detected in individual 5.

### 3.2. Description of Megalomeronts

**Figure 3:**
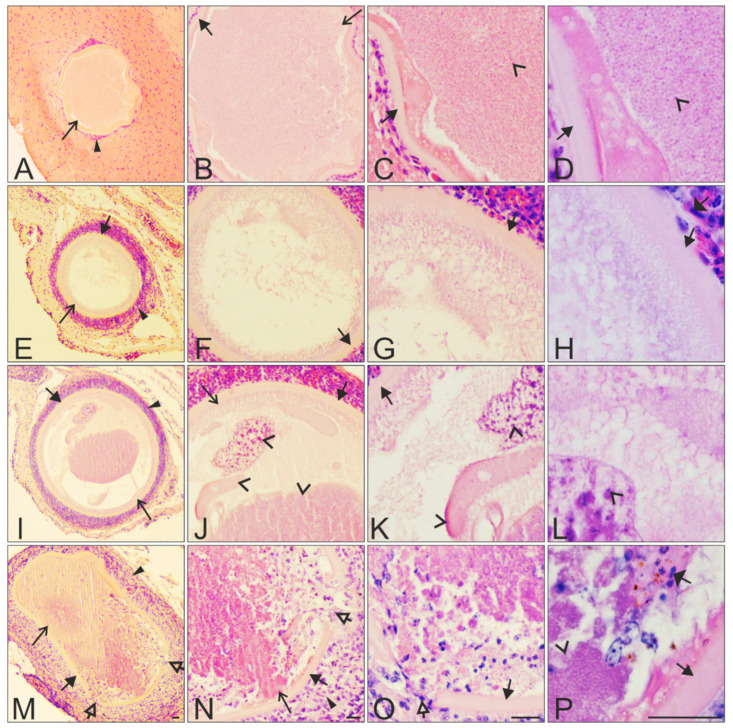
Megalomeronts of *Haemoproteus pastoris* (lineage hLAMPUR01) in the cerebellum of the brain (**A**–**D**) and esophagus (**E**–**P**) of naturally infected Common starlings *Sturnus vulgaris*. Note the prominent capsular-like wall covering each megalomeront (**B**–**P**). The megalomeront in the brain was mature and overfilled with completely developed merozoites (**C**,**D**). Images **E**–**L** show two different histological sections of the same megalomeront, which was cut at different depths; as this megalomeront likely was a roundish body in 3D, the parasite in image I was bigger than in image E due to the location of the former section being closer to the center of the parasite. Note that developing cytomeres were present in section I (located closer to the center of the parasite) but not in section E (located closer to the periphery of the parasite), indicating uneven cytomere location during their development within the same megalomeront. It is possible that nuclear division started first at the center of the roundish megalomeronts (section I), and while the parasite grew, megalomeront division continued until the merozoites reached the extremities of the megalomeronts (section E). Images M–P show a mature ruptured megalomeront containing mature merozoites and infiltration of blood cells inside the megalomeront. Each megalomeront was shown at four different magnifications: **A**, **E**, **I**, **M** ×100; **B**, **F**, **J**, **N** ×200; **C**, **G**, **K**, **O** ×400, and **D**, **H**, **L**, **P** ×1000. Simple arrows (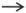)—megalomeronts. Filled-black triangle arrows (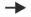)—capsular-like wall. Contoured-black triangle arrows (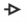)—rupture of the capsular-like wall. Flat triangle arrows (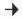)—red blood cells inside or outside the megalomeront. Triangle arrowheads (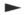)—deformed adjacent tissue cells suppressed by the megalomeront. Simple arrowheads (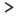)—merozoites. All scale bars = 20 μm.

**Figure 4:**
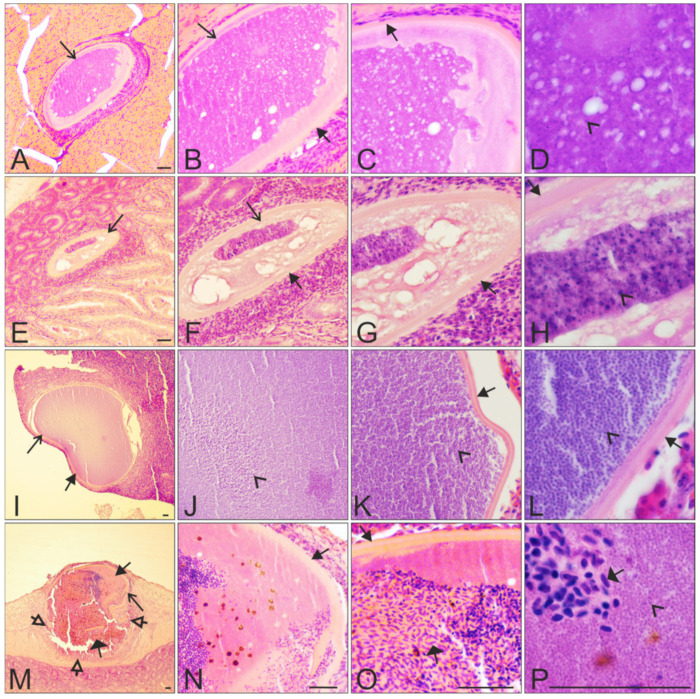
Megalomeronts of *Haemoproteus pastoris* (lineage hLAMPUR01) in the gizzard (**A**–**D**), the intestine (**E**–**H**,**M**–**P**), and the pancreas (**I**–**L**) of naturally infected Common starlings *Sturnus vulgaris*. Note the prominent capsular-like wall covering each megalomeront (**B**,**C**,**F**–**H**,**K**,**L**,**N**,**O**), the oval-shape of two megalomeronts (**A**,**E**), and the big size-difference between developing and mature megalomeronts (**A**,**E** vs. **I**,**M**). Megalomeronts were seen at different stages of development: E–H growing immature, **A**–**D** growing maturing, **I**–**L** completely mature, and **M**–**P** ruptured mature at the stage of degeneration. The megalomeront shown in **E**–**H** seemed to present cytomeres at two degrees of maturation, with the more mature merozoites in dark-purple color (**H**) while the rest of the megalomeront remained light pink and showed a less-developed structure; this could indicate an asynchronous development of cytomeres within the same megalomeront. Megalomeronts with a ruptured wall (**M**) showed marked infiltration of blood cells (**M**–**P**), with still some merozoites present (**P**). Each megalomeront is shown at five different magnifications: **I**, **M** ×40; **A**, **E**, ×100; **B**, **F**, **J**, **N** ×200; **C**, **G**, **K**, **O** ×400, and **D**, **H**, **L**, **P** ×1000. Simple arrows (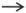)—megalomeront. Filled-black triangle arrows (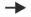)—capsular-like wall. Contoured-black triangle arrows (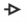)—rupture of the capsular-like wall. Flat triangle arrows (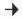)—red blood cells invading the megalomeront. Simple arrowheads (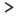)—merozoite. All scale bars = 40 μm.

**Figure 5:**
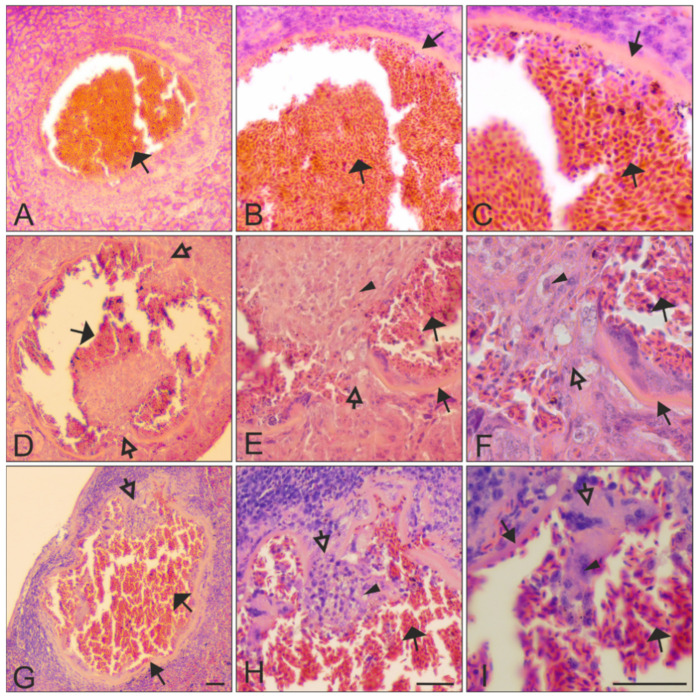
Ruptured mature megalomeronts of *Haemoproteus pastoris* (lineage hLAMPUR01) in the pancreas (**A**–**C**), the intestine (**D**–**F**), and the spleen (**G**–**I**) of naturally infected Common starlings *Sturnus vulgaris*. Note that the prominent capsular-like wall was still present after megalomeront maturation and release of merozoites (**B**,**C**,**E**–**I**), resulting in maintenance of a prominent space, which was invaded with numerous erythrocytes and other blood cells (**A**–**C** vs. **D**–**I**). Parasites were shown at three different magnifications: A, D, G ×100; B, E, H ×200, and C, F, I ×400. Filled-black triangle arrows (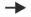)—capsular-like wall. Contoured-black triangle arrows (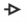)—rupture of the capsular-like wall. Flat triangle arrows (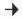)—red blood cells invading the megalomeront. Triangle arrowheads (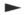)—other cells invading the megalomeront. All scale bars = 40 μm.

No meronts were seen in the histological sections of the five individuals, while megalomeronts were found in four of the five examined individuals (Table 1). Each megalomeront was surrounded by a prominent capsular-like wall, the thickness of which varied depending on the size of the parasites (Figure 3, Figure 4 and Figure 5). In total, combining all megalomeronts found per organs in all investigated starlings, the parasites were seen in the brain (1 parasite found in the cerebellum, Figure 3A–D), kidneys (1), lungs (1), intestine (13 in the mucosa and the muscularis, Figure 4E–H,M–P and Figure 5D–F), pancreas (3, Figure 4I–L and Figure 5A–C), esophagus (3 in the epithelium of the mucosa and the muscularis, Figure 1E–P), spleen (5, Figure 5G–I), gizzard (3 in the mucosa and the muscularis, Figure 4A–D), and the trachea (1). Megalomeronts were of similar morphology in all organs, however they were not seen in the same organs in each infected bird individual (Table 1). It is important to note that each megalomeront was considered as one entity, so if different sections of the same megalomeront were visualized, the parasite was still counted as the same one. For example, images in Figure 3E–L represent one megalomeront from two different cuts, and it was thus counted as one for the count in Table 1. As such, it is difficult to assess in which part of the megalomeront the section was performed if one preparation was available. In other words, it is difficult to know if it was cut more in its center or closer to the periphery, except when consecutive sections were performed as in Figure 3E–L. This is why, mainly, the biggest diameter of megalomeronts can be estimated as a factual parasite size feature. As megalomeronts seemed to be most often of a roundish shape, the observed smaller parasites on flat histological cuts may have been growing ones, but also sections of the parasite made closer to its periphery.

The majority of megalomeronts were of a roundish shape when growing (Figure 3A–L), with reported minimum size of 120 μm and a maximum size of 520 μm in diameter. Oval-shape megalomeronts were also observed among growing parasites, but such forms were only seen in the gizzard and the intestine probably due to the orientation of the muscle fibers in the latter organs (Figure 4A–H). The maximum length and width were 380 and 167 μm, respectively, for the oval megalomeronts. In growing megalomeronts, developing cytomeres were seen located unevenly inside the megalomeront, with numerous cytomeres present in one part of the parasite and fewer in other parts of the same parasite (Figure 3E–L and Figure 4A–H). The capsular-like wall was prominent, measuring between 2 and 12 μm in width at different sections.

One large, fully mature, and non-ruptured, bean-shaped megalomeront was observed in the pancreas of one bird individual; it measured 800 μm in its maximum length and about 560 μm in its width (Figure 4I–L). This parasite was overfilled with myriads of mature merozoites (Figure 4J–L), pressing against the capsular-like wall, which was up to 8 μm in width.

Several mature ruptured megalomeronts were found (Figure 3M–P, Figure 4M–P, and Figure 5; Table 1). They were mainly big and roundish bodied (570 μm in maximum diameter) with a still-visible capsular-like wall, which measured between 3 and 14 μm in width. However, the capsular-like wall was seen ruptured in one to several places (Figure 3M–O, Figure 4M, and Figure 5D–I), allowing erythrocytes and other cells to enter the megalomeront, while the merozoites leaked out (Figure 3M–O, Figure 4M, and Figure 5D–I). Finally, a structure full of blood corresponding to a former megalomeront, but still surrounded by a capsular-like wall, was observed in several organs (Table 1) and represented a stage of the degeneration of the megalomeront, as all merozoites had already escaped and only the host response was visible (Figure 5A–C). These parasites were mentioned as ‘ruptured’ in Table 1.

## 4. Discussion

The major outcome of this study was the discovery of exo-erythrocytic stages (megalomeronts) of *Haemoproteus* parasites developing in the brain of its avian host (Figure 3A–D). This indicates formerly underestimated pathology during avian haemoproteosis. Former studies observed megalomeronts of *Haemoproteus* species in the heart [11,16,19,20,25,27], intestine [22], kidneys [18,22,26], liver [17,18,26,36], lungs [19,20,26,36], muscles [10,11,13,15,16,19,21,23,27], gizzard [12,20], and spleen [10,26]. In this study, megalomeronts of *H. pastoris* (lineage hLAMPUR01) were observed in nine organs of naturally infected Common starlings, including the brain of one individual (Table 1). This finding is worth attention in future research aiming to better understand mechanisms of virulence during avian haemoproteosis.

It is important to note that the possibility that the found megalomeronts could belong to other blood parasites species can be ruled out due to the following observations. First, 19 Common starlings were examined microscopically, and other blood parasites were not seen, except for *Lankesteralla* sp. in bird no. 3. Second, the primers used for molecular barcoding could detect *Plasmodium* and *Haemoproteus* infections. Only one *Plasmodium relictum* infection was detected by PCR-based testing in one starling (individual 5). The life cycle of *P. relictum* was investigated experimentally, and megalomeronts were absent not only in this malaria parasite species but also in all described *Plasmodium* species in all groups of vertebrates [1]. *Plasmodium* parasites, which were reported before in Common starlings [3], do not develop megalomeronts but meronts [37], the structure and development of which differ from megalomeronts remarkably. *Leucocytozoon* species can be readily distinguished due to the presence of the markedly enlarged host cell nuclei [38,39], which is not the case for *Haemoproteus* species, and this character was absent in all found megalomeronts. Importantly, in all examined *H. pastoris* infected birds, we found the same lineage, morphotype, and megalomeronts of the same morphology. This repetition readily showed *H. pastoris* infection.

It is worth noting that exo-erythrocytic stages have been reported before in the brain of birds during other haemosporidian infections. Several species of *Plasmodium* sp. parasites produce phanerozoites in the brain [1,37,40,41,42], and some species of *Leucocytozoon* sp. develop megalomeronts in this organ as well [13,43,44]. Interestingly, both during *Plasmodium* and *Leucocytozoon* infections, exo-erythrocytic stages can be found all over the body of hosts as they develop in non-specialized cells, which are also present all over the body, i.e., endothelial cells lining capillaries (*Plasmodium* spp.) and macrophages (*Leucocytozoon* spp.) [13,39,42,43]. If the type of host cells during megalomerogony of *Haemoproteus* infections remains non-identified, it seems to be non-specialized cells as well, as nine different organs were seen to be parasitized during *H. pastoris* infection (Table 1), adding to the already-known parasitized organs for the other *Haemoproteus* species [13,22]. As megalomeronts are big in size and markedly deform the host cells and adjacent organ tissue, morphological characters cannot be used to determine the origin of the host cell. Further targeting studies using histochemistry and immunological methods are needed to answer this question.

The number of *Haemoproteus* species for which megalomeronts have been found, described, and linked to a lineage remains less than 10 (Figure 2) out of more than 150 identified species [2,45]. All studies reported big size of mature megalomeronts (usually >100 µm in biggest diameter) [13,20,21,22,23,24,25,26]. Megalomeronts of all described species of haemosporidians were covered with a capsular-like wall, and their maturation included a stage of formation of cytomeres [1,13,18,24,26]. This study supported these observations (Figure 3I–L and Figure 4A–H). Based on these two features, megalomeronts of *Haemoproteus* parasites are similar to megalomeronts of *Leucocytozoon* spp. In both parasite groups, the megalomeronts were covered by a hyaline wall of host origin [25,38], protecting developing parasites from the host immune system [13]. It is easy to distinguish megalomeronts of *Haemoproteus* and *Leucocytozoon* parasites due to presence of the enlarged host-cell nucleus in the latter (also called a central body) [38,39] and its absence in the former organisms [13,25].

The observed growing megalomeronts of *H. pastoris* showed asynchronous development of cytomeres, which were more developed in one part of the megalomeront (Figure 3I–L and Figure 4A–H) while looking less developed in other parts with regard to differentiation of nuclei (Figure 3E–H). This might have been due to different rates of merogony in different cytomere lobules of the parasite. Shown in Figure 3I–L, the parasite showed three different levels of maturation inside the same megalomeront in one section; this was visible due to differing intensity of the nuclear material staining; another section of the same megalomeront did not show cytomeres (Figure 3E–H). Another hypothesis would be that this under-developed part might actually not have been parasite material but belonged to the host cell itself. If we take the example of *H. minutus* investigated with in situ hybridization [25], the paler-outer structure present inside the wall did not react to the parasite probes, indicating its host origin. However, this would need more investigation as no such reports have been addressed for haemosporidians. Both these hypotheses might be tested by the combination of two approaches. First, experimental *Haemoproteus* infection, which would provide an opportunity to access development of megalomeronts in dynamics (for methods see [27,46]), and second, application of specific RNA probes, which would distinguish host and parasite structures (such as in situ hybridization, for methods see [25]).

Similar differences in color and cytomere structure could also be seen in the oval megalomeronts present in the intestine; in this parasite, better-differentiated merozoites were visible in one cytomere and less developed in another one, and their space occupation inside the wall also differed (compare Figure 4A–D vs. Figure 4E–H). It is worth mentioning that the organization of cytomeres within the megalomeront could have been parasite species-specific. Mainly, readily visible differences in cytomere morphology have been observed between the investigated *Haemoproteus* species thus far. Overall, megalomeront structure shows more differences than similarities between *Haemoproteus* species [13,20,21,26]. Among the distinctive features of megalomeronts, the following are readily distinctive: clustering of megalomeronts in tightly closely adjacent groups in *H. minutus* and *H. asymmetricus* [20,25]; development of megalomeronts only in tissues of the skeletal muscles (*H. mansoni, H. velans, H. syrnii*) and gizzard (*H. sacharovi*) [12,21,23,27]; presence of distinct separately located individuals cytomeres (*H. passeris*) [36]; grouping cytomeres separated from each other by septa (*H.* sp.) [24]; cytomeres interconnected (*H. majoris*) [22,26], or grouped cytomeres in tighter masses (*H. pastoris*) (Figure 3 and Figure 4).

Interestingly, the asynchronous development was seen both within (i) the individual megalomeronts in relation to cytomeres formation (see above paragraphs), and within (ii) the individual host in relation to different stages of parasite maturation (Table 1). Several stages of megalomeront development were observed in the same host—from growing (Figure 3A–L and Figure 4A–H), to mature (Figure 4I–L), and fully ruptured megalomeronts (Figure 3M–P, Figure 4M–P, and Figure 5A–I). This is similar to the development of *Haemoproteus mansoni* (syn. *Haemoproteus meleagridis*) megalomeronts, which were studied during an experimental infection [27] and were observed from early growing forms to mature and ruptured forms in different individuals of the same host species. Taking into account the four examined naturally infected different Common starling individuals (Table 1), the pattern was similar to these experimental data. Mainly, the completely matured megalomeronts were far bigger than the growing ones (Figure 4I–L vs. Figure 3E–L, Figure 4A–H), and their inside space was fully packed with mature merozoites (Figure 4I–L); the ruptured megalomeronts had their surrounding wall ruptured in one to several places and its general outline appeared a bit wavier (Figure 3M–P, Figure 4M–P and Figure 5) [10,11,27]. In other words, even within one individual host, different stages of growth of megalomeronts could be found; they did not develop synchronously (Table 1, Figure 3, Figure 4 and Figure 5). This was also previously observed in *H. majoris*, in which megalomeronts were more mature in the kidneys than in the intestine [22].

Asynchronous development of exo-erythrocytic stages (meronts and megalomeronts) have been reported in many *Plasmodium* and *Leucocytozoon* species due to different times of sporozoite penetration in host cells and also the presence of several generations of merogony [1,47,48]. The latter remains insufficiently investigated in *Haemoproteus* parasites [49]. Due to asynchronous development and maturation of merozoites, gametocytes also develop asynchronously, and this likely is important for transmission and infection of vectors [1].

Research on exo-erythrocytic stages of *Haemoproteus* parasites remains scarce, nonetheless, the available limited data suggest that phylogenetically distant species likely have different megalomeront morphology, as discussed above (Figure 2). However, parasites of well-supported phylogenetic lineage clades have similar megalomeront morphology and location, as is the case in different lineages of *H. majoris* [22,26]. This indicates that phylogenies based on cyt *b* partial genes might be used to predict megalomeront morphology and location in still-non-investigated parasites. Further studies are needed to prove this hypothesis.

The parasitemia intensity in all dissected bird individuals was different and relatively high (Table 1). Interestingly, reports of megalomeronts were not directly related to parasitemia intensity. For example, in bird no. 4 (Table 1), parasitemia was higher than in birds nos. 1–3 but megalomeronts were not seen in no. 4. Furthermore, the four megalomeront-positive individuals had different parasitemia intensity, but megalomeronts were found in a similar number of organs in all birds (three to four positive organs per individual, Table 1). *H. majoris* megalomeronts were found in different host species in two to four different organs [22,26], *H. minutus* in three organs [20], and *H. syrnii* and *H. velans* in one organ [21,23]. These data show that intensity of megalomeronts might have been low and they might have been difficult to find in histological sections even during high parasitemia; this should be taken into consideration during planning of histopathology research using naturally infected birds.

Megalomeronts of *H. pastoris* were mostly roundish, but also showed variability in size and shape, depending on the organ in which they were found and the stage of their development. The majority of observed growing megalomeronts were of a roundish shape (Figure 3A–L), and a few were of an oval-shape, but the latter were only seen in the gizzard and the intestine (Figure 4A–H). This is reminiscent of the young and small *H. majoris* (hPHSIB1) megalomeront found in the intestine of a wood warbler (*Phylloscopus sibilatrix*); it was found to be more oval than roundish in shape [22].

Megalomeronts have thus far always been reported surrounded by a capsular-like wall, which is of host origin [25], but it is still unclear of which host cells and structures it is composed [13,20,21,22,23,24,25,26]. The capsular-like wall, which surrounds the megalomeront, seems to be a malleable structure that can extend with the development of the megalomeront, fitting the parasite size and its location in organs, as its thickness was similar within reported variation in all observed megalomeronts of the same species [20,25,26,45]. It also showed a rigidness as it was still present after the maturation and burst of the megalomeront, keeping its place even after rupture (Figure 3, Figure 4 and Figure 5).

Indeed, the presence of the relatively rigid wall allowed the visualization of the fully ruptured megalomeront, from which merozoites escaped (Figure 3M–P, Figure 4M–P, and Figure 5). These merozoite-empty megalomeronts were full of red blood cells (Figure 5A–I). Due to the capsular-like wall, the ruptured megalomeronts partly maintained their shapes. As the wall surrounding growing megalomeronts is thick, entire, and is of host origin [25], no inflammatory reaction was observed around the parasites before the burst of the megalomeront. In fact, former studies also did not report the presence of inflammatory reaction around megalomeronts [13,21,24]. However, after the rupture of the wall, the merozoites came into contact with the host tissues, and a host response appeared. The latter was readily visible due to development of bleeding and prominent blood portions filling the megalomeront through the parts where the wall had broken (Figure 3M–P, Figure 4M–P, and Figure 5D–I) [10,11]. Hemorrhage-related processes likely are important pathologies during *H. pastoris* infection and might occur in haemoproteosis caused by other *Haemoproteus* spp. From this point of view, avian haemoproteosis might be similar to *Leucocytozoon (Akiba) caulleryi* hemorrhages caused in domestic chicken [47,50] and, thus, worthy of experimental research. In the case of *H. pastoris*, completely mature and ruptured megalomeronts were found in the brain, the pancreas, the intestine, the esophagus, and the spleen.

The range and timing of megalomeront development in different organs remain to be investigated. It might be that (i) some organs are preferred for megalomeront development at first, and later they develop in other organs, (ii) or they develop faster in some organs, (iii) or the cells they infect are not equally available in all organs, (iv) or other factors which remain unclear. Experimental research would be helpful to answer these questions, but remains rare for *Haemoproteus* spp. [10,27].

Depending on the organ and tissue where the megalomeronts develop, and also the parasite size, we observed a different amount of tissue cells that surround the parasite. These cells were arranged around the capsular-like wall as a thick layer, which was well recognizable in stained histological preparations (Figure 3, Figure 4A,E,I,M, and Figure 5A,D,G). The layer consisted of cells adjacent to the megalomeronts, which were pushed while the megalomeront grew bigger. Few cells seem to be gathered around megalomeronts in the muscles and heart [20,21,23,24,25], while in parenchyma-rich organs (liver, lungs, spleen, and kidneys) more cells were observed around megalomeronts [22,26]. This showed marked pressure, which megalomeronts produce within organs, and might be important in disease pathology, which needs further research.

This study did not provide certain information about how the reported infections might influence bird health, as the investigated Common starling individuals were wild-caught and dissected within several hours after catching once the parasitemia was confirmed. However, it was clear that the birds were leading active life in wildlife, at least for some time, since they were flying and entered the traps themselves. Presence of high parasitemia and pathological damage in organs by the presence of megalomeronts suggest that parasitized Common starlings might be ill, but still able to fly. Experimental observations are needed for better understanding of pathogenicity during avian haemoproteosis, but such studies are rare [10,12,27]. Some experimental observations showed that birds severely infected with *Plasmodium* parasites often experience sudden mortality: they look active in the evening but are found dead in the morning next day [1,37,40]. Similar events might occur during severe haemoproteosis, particularly during damage of the brain that can cause cerebral ischemia resulting in cerebral paralysis. Except rare observations on dead bird individuals, which were monitored in rehabilitation centers, zoos, and also a limited number of experimental observations [20,21,23,24], the knowledge about what might happen to wild-caught individuals because of haemosporidian infection remains limited. Several studies have reported hemorrhages, multifocal necrosis, and macroscopic organ changes (enlargement, changes of color), which were seen in *Haemoproteus* spp.-infected birds at necropsy [20,21,22,23,24].

The only *Haemoproteus* species recorded in the Common starling was *H. pastoris*, with only one lineage hLAMPUR01 reported (Table 2). This species and lineage have only been found in three species of birds, all belonging to the Sturnidae. Furthermore, only the hLAMPUR01 lineage was found in two of the bird species (Table 2). This indicates that this parasite might be specific to a few species of the Sturnidae. It is possible to speculate that adaptation to develop in birds of the Sturnidae provides opportunities to explore many organs for exo-erythrocytic development of *H. pastoris* (Table 1). If this hypothesis is correct, the same occupation of many organs might be expected in other host-specific *Haemoproteus* parasite lineages. In this regard, it is worth mentioning that *H. majoris* lineages parasitize several Paridae, Sylviidae, and Turdidae species among other avian hosts, and would thus be of broader specificity than *H. pastoris* [22,26,51]. Megalomeronts of *H. majoris* preferably developed in kidneys in all examined avian hosts [22,26]. It is also worth mentioning that other *Haemoproteus* parasites are found in the same host as *H. majoris* (MalAvi database, [3]), and thus, competition might happen during the development. It seems that a broad vertebrate host specificity of a *Haemoproteus* lineage might be related to a more specialized development of the parasite in certain groups of cells and organs. However, the available information on exo-erythrocytic development remains premature even for preliminary conclusions. Further accumulation of data on exo-erythrocytic development of avian haemoproteids is needed.

## 5. Conclusions

This study discovered and described megalomeronts in *H. pastoris*. For the first time, it was shown that the brain can be parasitized by megalomeronts during avian haemoproteosis. This finding broadens understanding of pathologies caused by avian *Haemoproteus* species and calls for further pathology research during these common and widespread bird infections. Hemorrhage-related processes likely are important pathologies during *H. pastoris* infection and might occur in haemoproteosis caused by other *Haemoproteus* species once megalomeronts mature and burst out merozoites. This issue is related to bird health and worth targeting in experimental research. Asynchronous development is a characteristic feature of *H. pastoris*, with asynchronous development of cytomeres inside individual megalomeronts and different megalomeronts in the same individual host. The mechanism of asynchronous development of cytomeres in individual megalomeronts remains unclear and worthy of attention for better understanding exo-erythrocytic development in haemosporidian parasites. The available information shows that structure of megalomeronts in all investigated *Haemoproteus* species is different, and these differences are supported by phylogenetic analysis, indicating the important value of this character for future taxonomy and *Haemoproteus* parasite biodiversity research.

## Figures and Tables

**Table 1:** Intensity of parasitemia and megalomeront-positive organs seen in the five dissected naturally infected Common starlings (*Sturnus vulgaris*). The number of visualized megalomeronts in histological sections (in parenthesis) and the stage of their development are shown. Note that reports of megalomeronts did not seem to be related to the intensity of parasitemia, as megalomeronts were not seen in individual no. 4, in which parasitemia was high (10%), but were present in bird no. 1, in which parasitemia was lower (1%). No megalomeronts were found in any of the individuals in the heart, liver, pectoral muscles, or the reproductive organs.

Individual Number	Parasitemia (%)	Brain	Intestine	Pancreas	Kidneys	Lungs	Esophagus	Spleen	Gizzard	Trachea
1	1	0 ^a^	growing (2)	0	growing (1)	0	ruptured (1)	growing (1) ruptured (3)	0	0
2	2	0	growing (1)	0	0	0	0	growing (1)	0	growing (1)
3	3	0	growing (4) ruptured (4)	growing (1) mature (1) ruptured (1)	0	growing (1)	0	0	growing (1)	0
4	10	0	0	0	0	0	0	0	0	0
5	26	mature (1)	ruptured (2)	0	0	0	growing (1) ruptured (1)	0	growing (2)	0

^a^ Organ examined, but megalomeronts were not found.

**Table 2:** Molecular records of *H. pastoris* (lineage hLAMPUR01) and non-identified to species level closely related lineage hLAMPUR02 in these avian hosts, according to MalAvi database [3], July 2021.

Parasite Species (Lineage)	Recorded	Bird Species (Family)	Country (Continent)
*H. pastoris* (hLAMPUR01)	1		Bulgaria (Europe)
	1	*Sturnus vulgaris* (Sturnidae)	Turkey (Asia)
	1		Iran (Asia)
*H. pastoris* (hLAMPUR01)	1	*Sturnus roseus* (Sturnidae)	Bulgaria (Europe)
*H. pastoris* (hLAMPUR01)	1	*Lamprotonis pupureiceps* (Sturnidae)	Gabon (Africa)
*H.* sp. (hLAMPUR02) ^a^	1	*Lamprotonis pupureiceps* (Sturnidae)	Gabon (Africa)

^a^ Genetic difference between the lineages hLAMPUR01 and hLAMPUR02 is 5 bp or 1.05%.

## Data Availability

The data, including parasite voucher preparations, are available on request from Nature Research Centre, Vilnius, Lithuania.
